# Radial diffusivity reflects general decline rather than specific cognitive deterioration in multiple sclerosis

**DOI:** 10.1038/s41598-022-26204-z

**Published:** 2022-12-16

**Authors:** Johan Baijot, Delphine Van Laethem, Stijn Denissen, Lars Costers, Melissa Cambron, Miguel D’Haeseleer, Marie B. D’hooghe, Anne-Marie Vanbinst, Johan De Mey, Guy Nagels, Jeroen Van Schependom

**Affiliations:** 1 AIMS Lab, Center for Neurosciences, Vrije Universiteit Brussel, Ke.2.13, Pleinlaan 2, 1050 Elsene, Brussels, Belgium; 2 Department of Physical and Rehabilitation Medicine, UZ Brussel, Brussels, Belgium; 3 Icometrix, Leuven, Belgium; 4 Department of Neurology, UZ Brussel, Brussels, Belgium; 5 AZ Sint-Jan, Brugge, Belgium; 6 National MS Center Melsbroek, Melsbroek, Belgium; 7 Department of Radiology, UZ Brussel, Brussels, Belgium; 8 St Edmund Hall, University of Oxford, Oxford, UK; 9 Department of Electronics and Informatics (ETRO), Vrije Universiteit Brussel, Brussels, Belgium

**Keywords:** Multiple sclerosis, Cognitive neuroscience, Diffusion tensor imaging

## Abstract

Advanced structural brain imaging techniques, such as diffusion tensor imaging (DTI), have been used to study the relationship between DTI-parameters and cognitive scores in multiple sclerosis (MS). In this study, we assessed cognitive function in 61 individuals with MS and a control group of 35 healthy individuals with the Symbol Digit Modalities Test, the California Verbal Learning Test-II, the Brief Visuospatial Memory Test-Revised, the Controlled Oral Word Association Test, and Stroop-test. We also acquired diffusion-weighted images (b = 1000; 32 directions), which were processed to obtain the following DTI scalars: fractional anisotropy, mean, axial, and radial diffusivity. The relation between DTI scalars and cognitive parameters was assessed through permutations. Although fractional anisotropy and axial diffusivity did not correlate with any of the cognitive tests, mean and radial diffusivity were negatively correlated with all of these tests. However, this effect was not specific to any specific white matter tract or cognitive test and demonstrated a general effect with only low to moderate individual voxel-based correlations of <0.6. Similarly, lesion and white matter volume show a general effect with medium to high voxel-based correlations of 0.5-0.8. In conclusion, radial diffusivity is strongly related to cognitive impairment in MS. However, the strong associations of radial diffusivity with both cognition and whole brain lesion volume suggest that it is a surrogate marker for general decline in MS, rather than a marker for specific cognitive functions.

## Introduction

Multiple sclerosis (MS) is the most common inflammatory, demyelinating, and neurodegenerative disease of the central nervous system in young adults^1^. Between 40 and 70% of the persons with multiple sclerosis (PwMS) suffer from cognitive impairment^2,3^, which has a detrimental effect on quality of life^4^ and coping mechanisms^5^, and cannot be accurately predicted by disease duration^6^.

Assessing cognitive impairment is difficult and time-consuming. At the same time, it is subject to learning effects, test–retest variability, and interrater variability. MR imaging has been explored to provide more objective and reliable biomarkers of the patient’s cognitive functioning^3,7^. Yet, correlations between cognitive test results and brain volume loss or lesion volume are weak (0.2–0.4)^8^. Additionally, atrophy as seen on MRI indicates substantial neuronal cell death, which means that the window of opportunity to prevent neuronal loss has passed. Therefore, alternative markers of cognition are being investigated. One option is to look at how the brain functions using electro- or magnetoencephalography or functional MR imaging. While promising results have been achieved^9,10^, we recently provided indirect evidence that the analysis of fMRI networks may be affected by vascular changes^11^.

Another option is to assess the brain’s microstructural integrity through diffusion-weighted imaging (DWI). By modelling the diffusion of water molecules along white matter fibre bundles through diffusion tensor imaging (DTI), several measures are calculated for each voxel. The parameters most assessed are Fractional Anisotropy (FA) and Mean Diffusivity (MD). A recent systematic review^12^ concluded that information processing speed (IPS) is correlated with DTI measures of several white matter bundles, mainly in the corpus callosum. However, many studies used the Paced Auditory Serial Addition Test (PASAT) to measure IPS. Because PASAT is poorly tolerated and the Symbol Digit Modalities Test (SDMT) is easier to administer, SDMT has replaced PASAT as the most commonly used cognitive task to assess IPS in the past decade^3^.

Comparing brain microstructure between subjects poses two problems: dealing with spatial anatomical variability and correcting for multiple comparisons. The field has evolved from lowering the significance threshold in voxel based analysis^13^ according to multiple comparisons methods, such as Bonferroni^14^, to cluster-based analysis with set thresholds^15^ and finally threshold-free methods, such as threshold-free cluster enhancement (TFCE)^16^. A recent review article by Manca et al.^12^ analysed brain processing speed in relation to DTI states. The authors showed that past literature presented a high variability because various older multiple comparisons approaches were used and thus results were inconclusive, creating a need for further research of the DTI parameters within MS. Other studies using TFCE^17,18^ had small sample sizes and limited scope by using only one DTI parameter or cognitive test. Meijer et al.^18^ did not experience these limitations but only investigated a dichotomization of the cognitive status (i.e., cognition impaired versus preserved), instead of studying separate cognitive domains.

In this study, we revisit the hypothesis that DTI parameters are correlated with cognition. Furthermore, by including cognitive tests that evaluate the most affected cognitive domains in MS, we will assess whether changes in DTI parameters are specific to certain cognitive domains. We perform a group analysis comparing the DTI parameters of 61 PwMS with 35 healthy subjects (HS), using tract-based spatial statistics (TBSS)^19,20^. Second, we assess each cognitive domain and its relation to the DTI parameters in our complete study sample throughout the whole brain, by combining the advantages of applying a TFCE as a cluster-based analysis and a voxel-based analysis, similar to Bernabéu-Sanz 2021^21^.

## Results

### Demographics, clinical, and neuropsychological data

Our study included 61 PwMS and 35 HS. The characteristics of each group are presented in Table 1. Age and sex were similar in both groups, but there was a statistically significant difference in terms of education level, fatigue, and depression. While both groups performed similarly on verbal and visual memory tests and the combined test for processing speed and attention, PwMS scored significantly lower on tests for processing speed, verbal fluency, and hand motricity.

**Table 1:** Demographics and neuropsychological scores of the PwMS and HS group.

	PwMS (n = 61)	HS (n = 35)	*p* value
Age in years (M ± SD)	47.5 (± 9.7)	48.4 (± 11.7)	0.68
Sex (Men/Women)	27/34	18/17	0.50
Education level in y (M ± SD)	14.2 (± 2.5)	15.4 (± 2.1)	**0.02**
Beck’s Depression Inventory (M ± SD)	11.4 (± 7.5)	5.8 (± 5.6)	**0.0003**
Fatigue Scale for Motor and Cognitive Functions (M ± SD)	63.9 (± 17.1)	33.9 (± 10.3)	** < 0.0001**
EDSS (Med [IQR])	3 [2–4]		
NHPT in peg/second (M ± SD)	0.41 (± 0.08)	0.45 (± 0.05)	**0.0011**
Disease duration in y (M ± SD)	15.4 (± 8.4)		
**Cognitive evaluation**			
SDMT (M ± SD)	48.6 (± 11.2)	54.8 (± 9.5)	**0.0075**
CVLT (M ± SD)	62.9 (± 10.6)	66.0 (± 7.2)	0.12
BVMT (M ± SD)	25.6 (± 7.3)	28.4 (± 5.6)	0.053
COWAT (M ± SD)	31.5 (± 9.2)	36.8 (± 8.4)	**0.0085**
Stroop (M ± SD)	40.7 (± 24.2)	32.4 (± 17.0)	0.10

*p* values were derived from two-sample t-tests, and from a Chi-square test for the variable sex.

*M* mean, *SD* standard deviation, *IQR* interquartile range, *y* years.

### Analysis of diffusion tensor parameters: group differences

**Figure 1:**
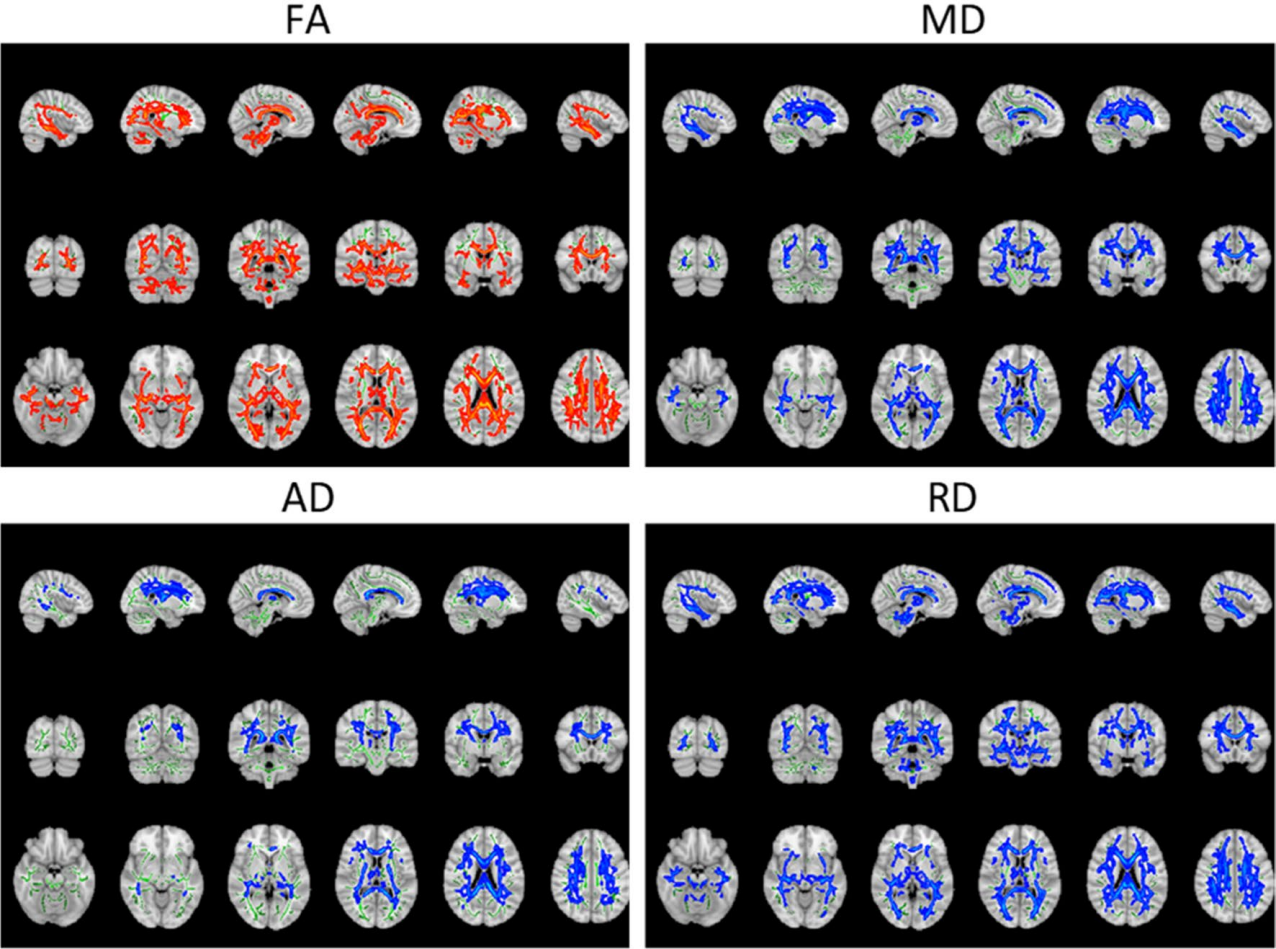
Tract-based spatial statistics nonparametric permutation inference results of comparing the diffusion tensor parameters of PwMS and HS. For better visualisation purposes the results are projected on top of a standard MNI image, with the assessed IIT-FA-skeleton shown in green and the significant outcomes thickened. Regions in red and blue respectively indicate a significant decrease or increase of the diffusion tensor parameter in PwMS compared to HS. For better visualization, the data can be visualized in 3D in the repository: https://neurovault.org/collections/LYEGOWBT/.

In a first analysis, we compared the diffusion tensor parameters of the PwMS group to those of the HS group; the results are illustrated in Fig. 1. PwMS showed a general decrease in fractional anisotropy (FA), across the whole white matter and an increase in mean diffusivity (MD), axial diffusivity (AD) and radial diffusivity (RD) in all regions. The results of the cluster analysis of the permutation testing are presented in Table 2. Only one large cluster was found for each of the diffusion tensor parameters.

**Table 2:** Parameters of the analysis of clusters of the permutation testing for each diffusion tensor parameter.

	Volume		Mean *p* value	Min *p* value
RD	43,606 mm^3^	39.4%	0.0072	< 0.001
FA	39,257 mm^3^	35.4%	0.008	< 0.001
MD	37,397 mm^3^	33.8%	0.0081	< 0.001
AD	21,999 mm^3^	19.9%	0.007	< 0.001

The parameters of the clusters listed are volume in mm^3^ and in % compared to the total WM tracts volume (of 110,750 mm^3^), mean *p* value and min *p* value.

### Correlation of diffusion tensor parameters with cognitive scores

**Figure 2:**
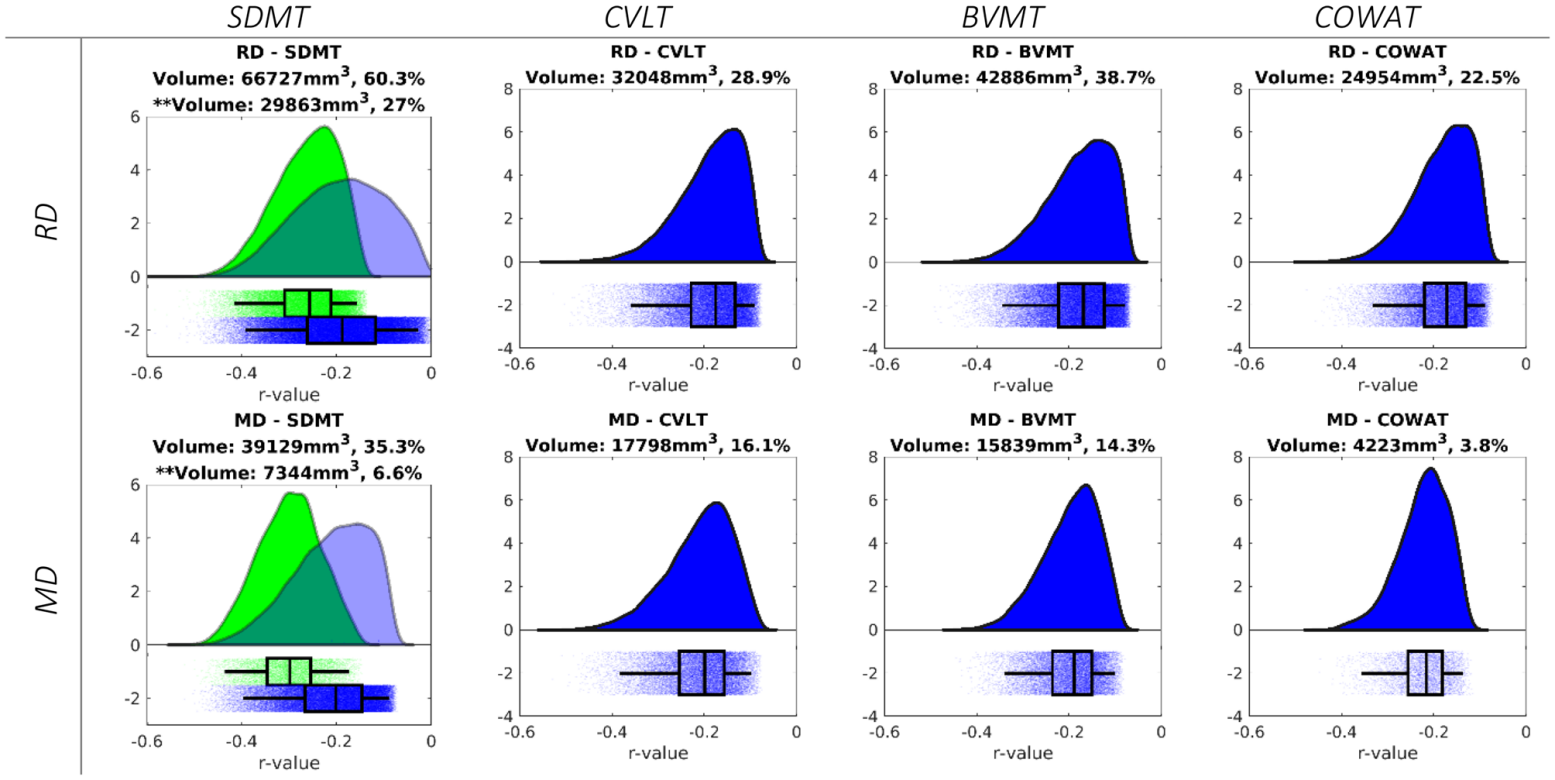
Distribution of r-values from the voxel-wise Pearson correlations between the diffusion tensor parameters and neuropsychological z-scores, for the significant voxels of the TFCE analysis. Results using the cut-off of 0.001 are shown in green and the corresponding volume is indicated with **. In blue we also show the results of cut-off 0.05 and its corresponding volume.

In a second analysis, we assessed the relation between the diffusion tensor and neuropsychological parameters. First, we identified clusters that were significantly correlated with the different cognitive tests through TBSS and TFCE. The visualisation of these intermediate results is available in an online repository (https://neurovault.org/collections/LYEGOWBT/) aligned to the MNI-space in Neuroimaging Informatics Technology Initiative (NIfTI) format. In the next step, the correlations between DTI parameters of the voxels within these clusters and the different cognitive parameters were assessed using the significant clusters as mask for the calculations, which is shown in Fig. 2. Finally, these correlations were evaluated using a white matter bundle atlas. There were no significant correlations between FA and AD and any of the assessed variables. MD and RD were correlated with the Symbol Digit Modalities Test (SDMT), California Verbal Learning Test-II (CVLT), Brief Visuospatial Memory Test-Revised (BVMT), and the Controlled Oral Word Association Test (COWAT) but only the SDMT correlation passed the correction for multiple comparisons. The strongest correlation for each white matter bundle is listed in Table 3.

**Table 3:**
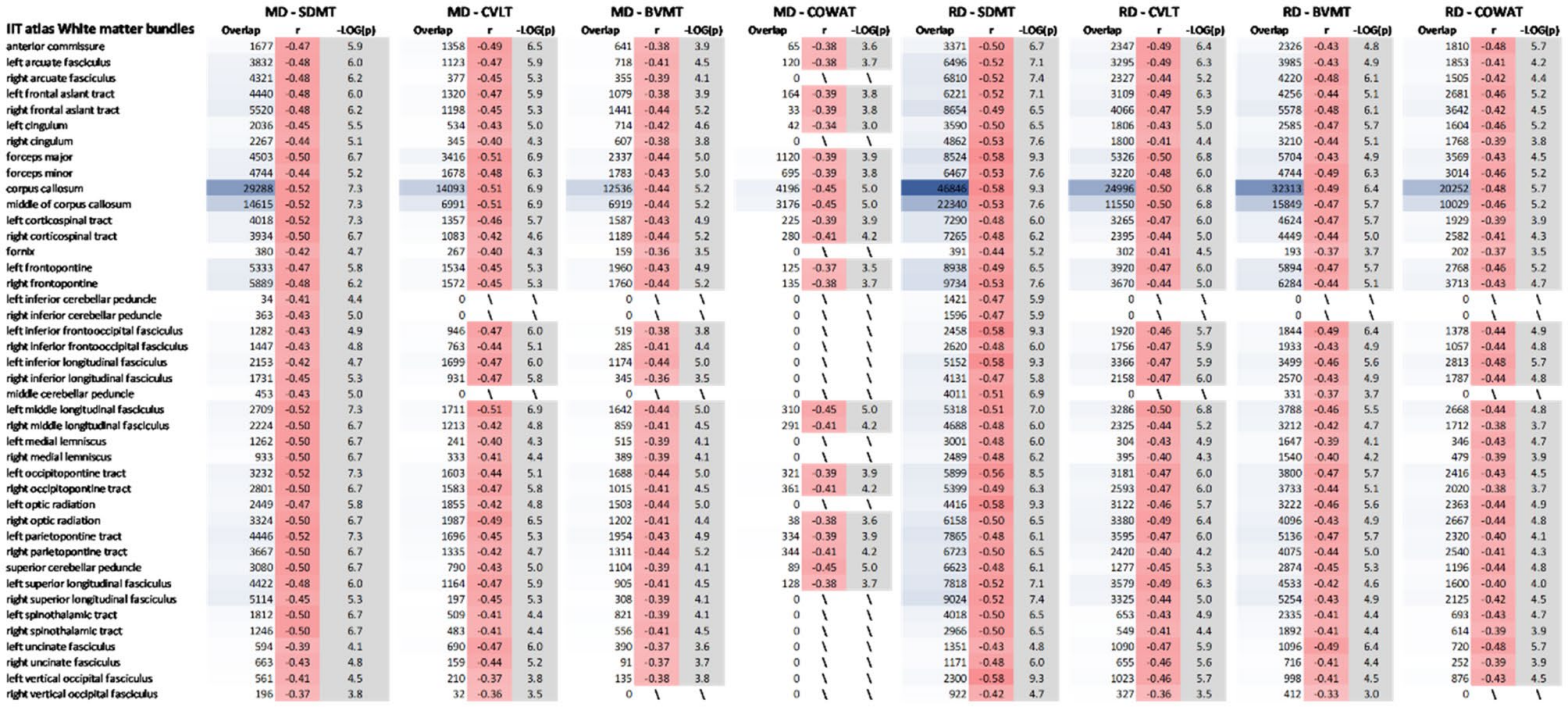
Strongest observed correlations between the diffusion tensor parameters and neuropsychological z-scores for each bundle of the IIT white matter bundle atlas.

Bundles presenting no significant correlations are indicated with a backslash, and a red (negative) and green (positive) colour palette is used to ease visual interpretation of the r-values strength and direction. The r column represents the strength of the correlation and the − LOG(*p*) shows the significance on the inverse logarithmic scale (the cut-off *p* value of 0.001 is − LOG10(*p*) = 3). The volume of the shared region between the considered cluster and the tracks is shown in mm^3^.

*MD* mean diffusivity, *RD* radial diffusivity, *BVMT* brief visuospatial memory test-revised, *COWAT* controlled oral word association test, *SDMT* symbol digit modalities test, *CVLT* California verbal learning test-II.

### Correlation of diffusion tensor parameters with volumetric parameters and EDSS

**Figure 3:**
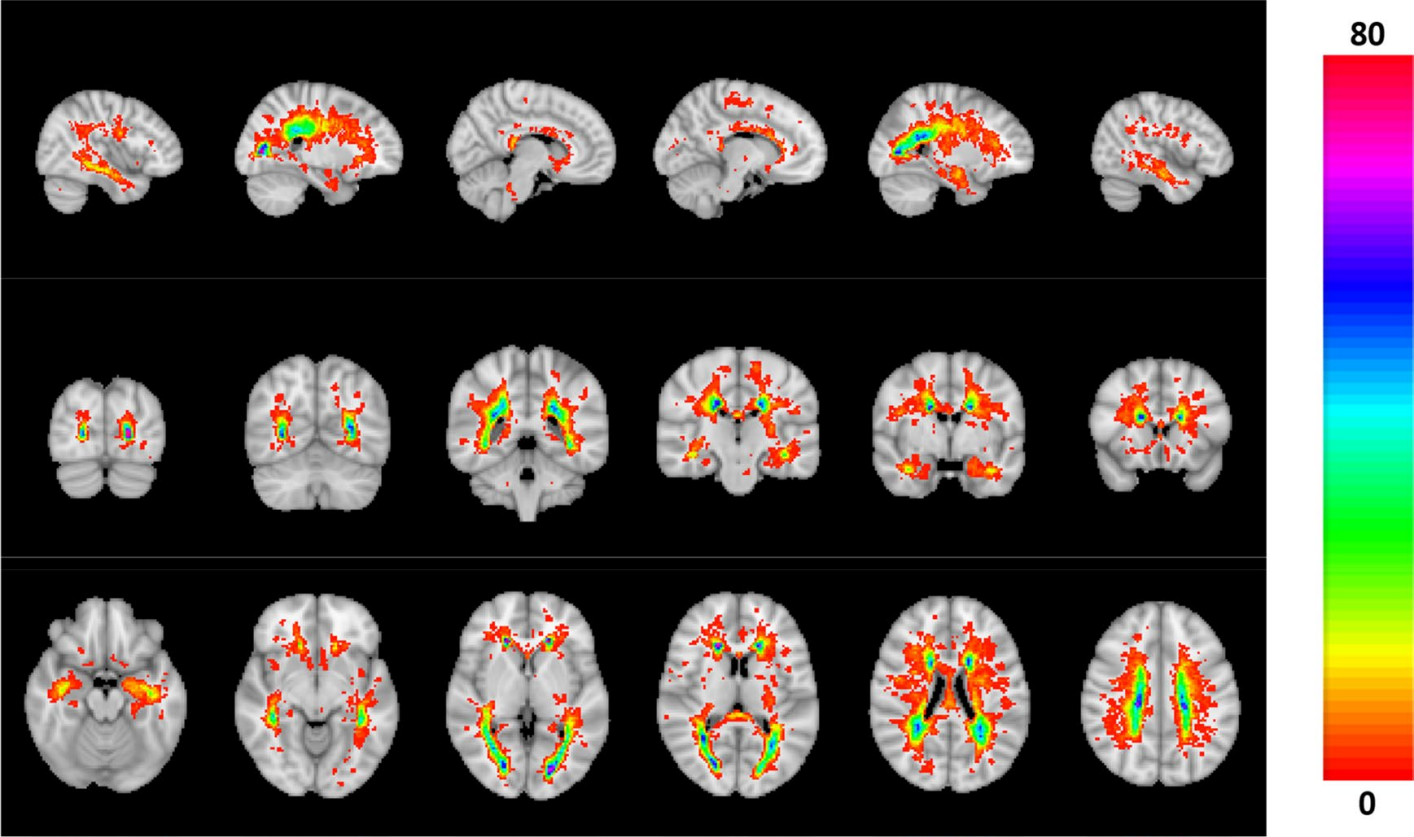
Map of the likelihood of lesions (FLAIR hyperintensities) in the MS group (in percentage).

Combining all T2 FLAIR hyperintensity lesion maps, we created a heat map to indicate the probability that a lesion was detected in a certain location in our dataset (see Fig. 3).

**Figure 4:**
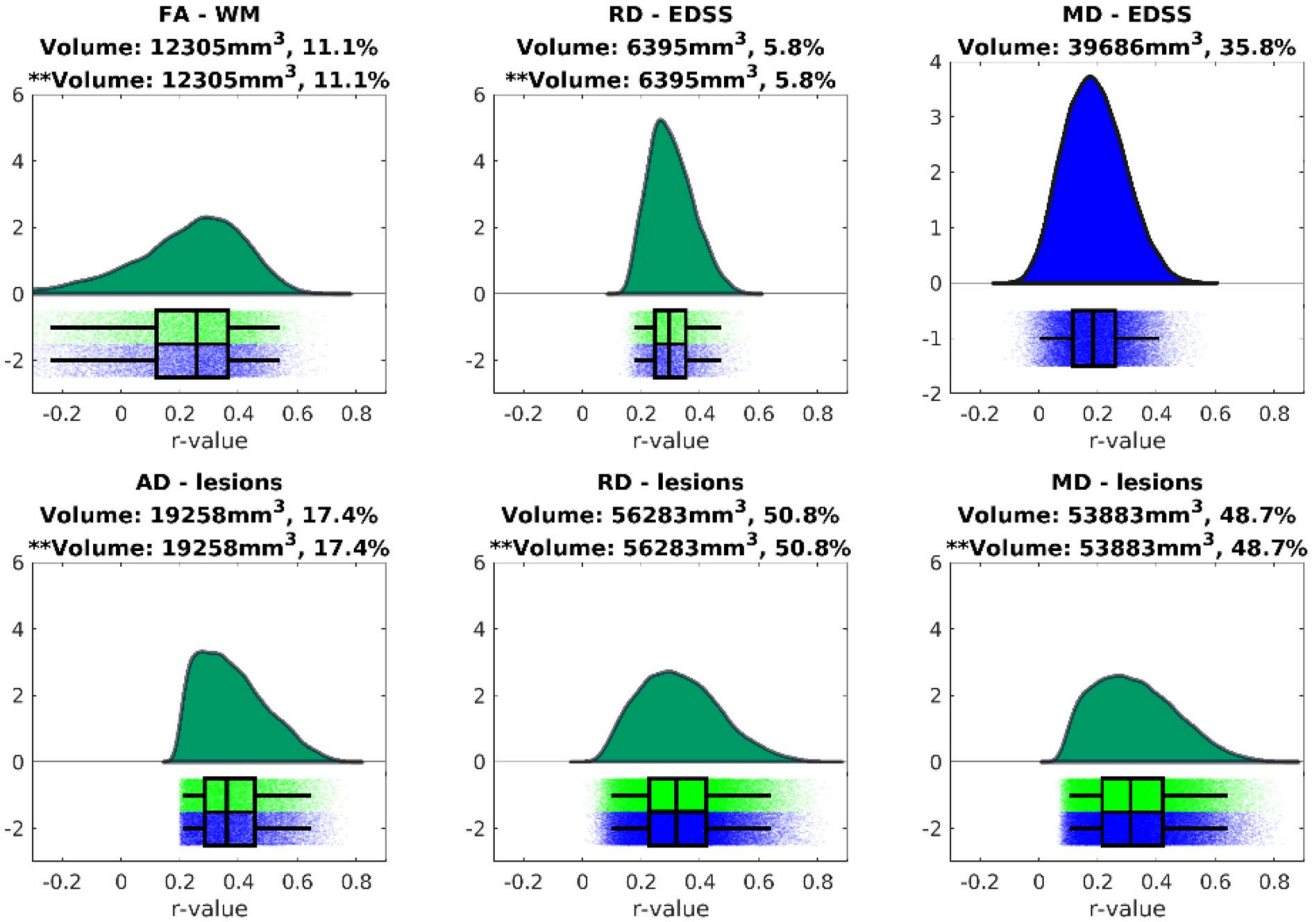
Distribution of r-values from the voxel-wise Pearson correlations between the diffusion tensor parameters and volumetric parameters and EDSS, for the significant voxels of the TFCE analysis. Results using the cut-off of 0.001 are shown in green and the corresponding volume is indicated with **. In blue we also show the results of cut-off 0.05 and its corresponding volume.

Furthermore, we assessed the association between tensor parameters and lesions volume, WM volume and EDSS. The intermediate results are provided in the online repository: https://neurovault.org/collections/LYEGOWBT/. The voxel-based correlations between lesions, WM volume, and EDSS and DTI parameters within these significant TFCE clusters are shown in Fig. 4. The strongest correlations for each white matter bundle from the IIT atlas are listed in Table 4. We found that only FA was significantly related to the white matter volume, while AD, MD, and RD were significantly associated with lesion volume. Also, MD and RD were significantly associated with the EDSS, but the correlations with MD did not pass correction for multiple comparisons.

**Table 4:**
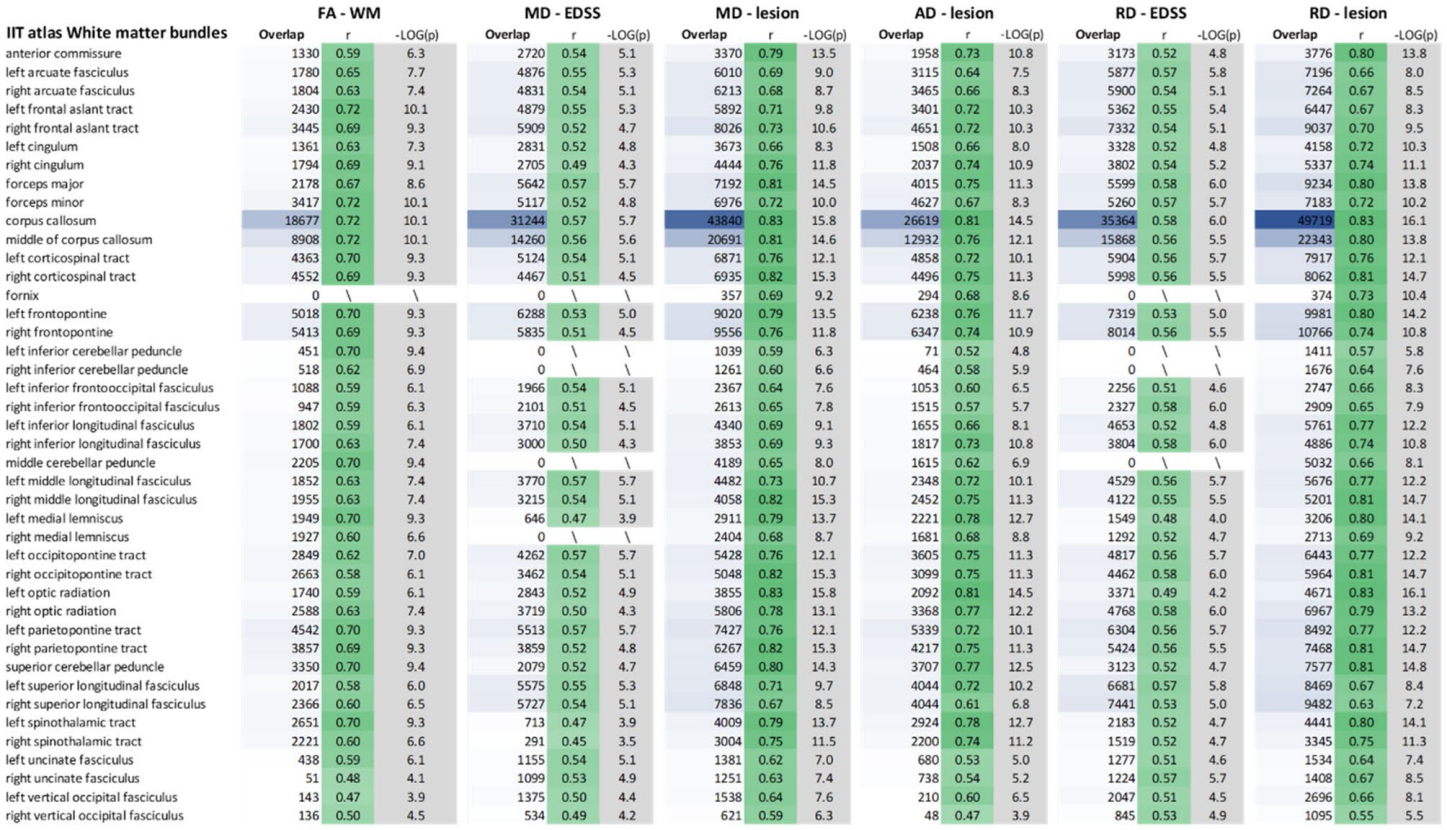
Strongest observed correlation in the PwMS group between diffusion tensor parameters and volumetric parameters and EDSS for each bundle of the IIT white matter bundle atlas.

Bundles without any significant correlations are indicated with a backslash and a red (negative) and green (positive) colour palette is used to facilitate visual interpretation of the R values. The r column represents the strength of the correlation and − LOG(*p*) shows the significance on the inverse logarithmic scale (cut-off *p* value of 0.001 is − LOG10(*p*) = 3). The volume of the shared region between the considered cluster and the tracks is shown in mm^3^.

*MD* mean diffusivity, *RD* radial diffusivity, *BVMT* brief visuospatial memory test-revised, *COWAT* controlled oral word association test, *SDMT* symbol digit modalities test, *CVLT* California verbal learning test-II.

## Discussion

In this paper, we compared DTI parameters between MS and HS and observed a significant decrease in FA in PwMS. This likely reflects a decrease in microstructural integrity^22,23^. Furthermore, there was an increase in AD and RD in PwMS compared to HS. These parameters have been related to demyelination in ex vivo^24^ and mouse studies^25,26^. Since MD can be expressed as a linear combination of RD and AD^23^, it is linked to the same biophysical properties and therefore also increased in PwMS. The associations between AD and RD and biophysical properties have been questioned as generalizations, in part because these parameters can be altered in pathological brain tissue without reflecting the organization of the underlying tissue. Numerical simulations of the DTI parameters with crossing fibres associated an increase in AD and RD with demyelination^27^. With axonal degeneration, we expect FA and AD to decrease while RD increases; with demyelination we expect FA to decrease, AD to be unaffected and RD to increase^28^. Despite the need for a careful interpretation, our findings indicate a clear pathology-induced alteration of the brain structure in PwMS compared to the HS. We observed a correspondence between the MS lesions map (available in Fig. 3) and regions showing significant group differences of the DTI parameters.

In a second step, we looked at our main research question, the relation between DTI-parameters and cognitive scores. On this subject, the literature was highly variable and inconclusive^12^. Bernabéu-Sanz 2021^21^ indicated thalamic atrophy as the main source of cognitive impairment in general and found associations between abnormalities in the occipital projection fibers of the corpus callosum and the SDMT in mildly disabled patients with relapsing–remitting multiple sclerosis (RRMS). Riccitelli 2019^29^ also studied RRMS and associated the PASAT and SDMT with widespread abnormalities in DTI in several regions, but the strength of these associations was not reported. Similarly, studies that compared cognitively impaired and cognitively preserved persons with MS (Meijer^18^ and Zhao^30^) or studies assessing a specific brain area (i.e., the thalamus in Benedict^31^ and corpus callosum in Sun^32^) have reported similar results. However, the question arises as to whether these associations are specific to information processing speed or reflect a more general (cognitive) decline.

We observed that MD and RD were correlated with the SDMT. Furthermore, the TBSS analysis showed one large significant cluster when correlations were found. This is in correspondence with Riccitelli et al.^29^, who found correlations between SDMT and MD in all bundles, except for the right cingulum, right fornix, and left posterior thalamic radiata. The association of SDMT with numerous pathways is not surprising. A functional MRI independent component analysis of an SDMT task has found that information processing speed is related to the visual network, cerebellum network, motor network, auditory network, visuospatial processing and reasoning network, and the default mode network^33^. At the voxel-level, we found low to medium correlations, below 0.6, between the DTI parameters and cognitive tests in the PwMS and HS group. The corpus callosum consistently shows the greatest overlap and a high correlation with cognition compared to the other tracts. Damage to the callosal tracts leads to dissociation of the connections between the hemispheres and has been associated with cognitive impairment in MS, as reported in functional connectivity studies^34,35^. Furthermore, diffusion tensor parameters in the corpus callosum have been consistently associated with processing speed^12,32^.

We did not observe any association between FA/AD and the different cognitive tests assessed through nonparametric permutation on the TBSS tracts. This is in line with previous studies, which reported weak^12,30^ or absent^12,32^ correlations. Other studies only found correlations with the PASAT^12^, but this test only has moderate sensitivity to cognitive deficits and has been replaced by the SDMT in clinical practice^3^. Some studies found correlations with other cognitive tests, but only in the corpus callosum^30,32^. Finally, a decreased FA has been observed in cognitively impaired PwMS compared to those with intact cognition^18^.

Based on functional connectivity studies, our expectation was that the different cognitive tests would show more distinctive correlation patterns with MD/RD, instead of a rather homogeneous correlation strength. SDMT, for instance, measures information processing speed and is linked to several brain regions (Silva et al.^36^ found 19 pairs of cortical regions in relation to the information processing network), while the CVLT measures verbal memory, which is predominantly associated with the left medial temporal lobe, the right hippocampus, and right frontal lobe on fMRI^37^. This discrepancy between our expected and observed findings could mean that MD/RD are markers of more general damage in MS, rather than specific markers of cognitive deterioration.

We examined the association of whole brain lesion volume and white matter volume with the tensor-based parameters. In this analysis much stronger correlations, with r values between 0.45 and 0.83, were observed for all bundles. Additionally, the brain regions that show significant correlations between MD, RD, AD and lesion volume are larger than the regions that showed significant correlations between MD, RD and all the cognitive tests. Considering that RD and MD show similar correlation patterns with cognitive tests, white matter, lesion volume, and EDSS scores, we suggest that MD and RD are plausible surrogate markers for the general decline of brain performance due to MS rather than specific markers for cognitive decline. Since AD was not correlated with any of the neuropsychological tests, the correlation between MD and the different cognitive tests seems to be driven by RD^27^. As such, RD appears to be of particular interest in assessing cognitive impairment in multiple sclerosis.

Several limitations must be considered: The two groups were matched for age and sex, but not for education level. Despite their lower education level, which is usually associated with a lower cognitive score^38^, the PwMS group did not perform significantly worse on 3 of the 6 cognitive tests (CVLT, BVMT, and Stroop). Repeating the analysis with a slightly smaller cohort of MS patients that was matched for education (*p* > 0.10) did not affect our results. All PwMS were recruited either in an academic hospital (UZ Brussel) or a reference centre (National MS Center of Melsbroek), where they regularly undergo cognitive testing, either for clinical evaluation or in the context of research. This could have resulted in a learning effect due to repeated test exposure^7^. This learning effect will decrease the sensitivity of our analysis. Additionally, our study has a cross-sectional design. A longitudinal study design on cognitive deterioration in MS could provide more insight into the associations between cognition and structural connectivity measures. Furthermore, medication and MS type were not considered in this study. Since we used a cross-sectional design, and therefore only looked at a fixed situation and not to the evolution, it could be neglected. For a longitudinal study, these aspects however cannot be neglected. Moreover, there are limitations inherent to the methods used for image processing and analysis: First, TBSS was designed to circumvent the partial volume effect, but the registration and projection of all subjects to one skeleton remains a critical step, where atrophy and lesions will inevitably worsen the sensitivity to construct the tract in PwMS^19^. As the projection depends on the morphology, any surrounding lesions or atrophy can introduce voxel misassignment. Second, the TFCE method includes a spatial smoothing before demonstrating the significance of clusters. Thus not guaranteeing the correlations of any voxel within the cluster. However, this should reduce false negative results on a voxel level. Finally, we used the maximum correlation as an approximation of the relative importance of the cluster. However, this intrinsically introduces a bias, namely, to potentially overestimate the importance of the cluster.

In conclusion, RD is generally considered a robust marker of demyelination and is strongly correlated with cognitive markers. Our results indicate that correlations with cognitive impairment are not limited to specific tracts, but rather affect all the white matter. Furthermore, these correlations are not specific to distinct cognitive domains but rather reflect the overall damage to the central nervous system caused by MS.

## Methods

### Compliance with ethical standards

Ethical approval was provided by the ethical committee: “commissie medische ethiek van MS Center Melsbroek” on 12 February 2015 and by the “medical ethics committee UZ BRUSSEL—VUB” on 25 February 2015 (B.U.N. 143201423263). Data analysis was carried out according to these study protocols and following the applicable local regulations. Written informed consent was obtained from all participants prior to inclusion.

### Data collection

The PwMS were recruited from the National MS Center of Melsbroek and the Universitair Ziekenhuis Brussel—VUB and the HS were recruited from hospital staff and acquaintances of the PwMS. All study participants were between 18 and 65 years old and were able to undergo MRI (absence of contraindications, eg pacemaker, prosthesis). Only patients with a diagnosis of definite MS according to the revised McDonald criteria^39^ and an Expanded Disability Status Scale score^40^ (EDSS) below 6.0 were included. HS were excluded if they suffered from any neurological condition or if they had first-degree relatives with MS.

In a timeframe of maximum 3 days, two testing sessions were organised, one to obtain demographical and clinical data as well as cognitive and other testing results and one to acquire a sequence of MR images. The demographical and clinical data included age, sex, education level and disease duration. Neuropsychological testing included the Brief international cognitive assessment for MS (BICAMS) test battery^41,42^ which consist of the Symbol Digit Modalities Test (SDMT, information processing speed)^41^, California Verbal Learning Test-II (CVLT, verbal memory and learning)^41^, and the Brief Visuospatial Memory Test-Revised (BVMT, visual memory and learning)^41^. The Controlled Oral Word Association Test (COWAT, verbal fluency)^43^ and the Stroop-test (Stroop, processing speed and attention)^44^ were also carried out. The cognitive scores were z-transformed for future analysis using the HS group as a reference. This allowed us to correct for age, education level and sex of the subjects, we refer to Costers et al.^42^ for more details.

### MRI acquisition

All scans were carried out at UZ Brussel on a 3T Philips Achieva scanner. The sagittal T1 weighted brain MRIs were acquired with the following parameters: field of view: 240 mm × 240 mm, 310 slices, voxel size: 0.5 mm × 0.5 mm × 0.5 mm, flip angle: 8°, repetition time (TR): 5.19 ms, echo time (TE): 2.30 ms. The FLAIR sequence was acquired with the following parameters: field of view: 240 mm × 240 mm, 320 slices, in-plane resolution: 1.0417 mm × 1.0417 mm, slice thickness: 1.12 mm, 0.56 mm space between slices, TR: 1650 ms, TE: 307 ms.

Diffusion-weighted images were acquired on the same scanner in the same session with 32 volumes with a non-collinear diffusion gradient and a b-value of 1000 s/mm^2^, one volume with b-value 0 s/mm^2^ (B0) and one volume with reverse-phase encoding. These images were acquired with the following parameters: in-plane field of view: 250 mm × 250 mm, 70 slices, in-plane resolution: 0.975 mm × 0.975 mm, slice thickness: 2 mm, TR: 5133 ms, TE: 95 ms.

### Image processing

MRtrix3^45^ and FSL^46–48^ software packages were used for processing and MATLAB 2019a was used for further statistical analysis. The diffusion-weighted images (DWI) were denoised^49 ^and the Gibbs-ringing effect^50^ was removed before estimating the susceptibility-induced field, based on the B0 and reverse phase-encoded image^51^. Correction for eddy currents^52^ and movement in the diffusion data was carried out using outlier replacement^53^ (using 3 standard deviations as a threshold due to the low number of acquired directions), the slice-to-volume motion correction^54^ (with 8 iterations and a degree of freedom of 8) and susceptibility-by-movement correction^55^. We performed bias field correction through the N4 BiasFieldCorrection algorithm from the advanced normalization tools (ANTs) toolkit^56^. This algorithm is an improved variation of the nonparametric nonuniform normalization retrospective bias correction algorithm. From these images, the diffusion tensor was estimated using the weighted linear least-squares estimation^57^. Different maps of the following tensor-derived parameters were created: fractional anisotropy (FA), mean diffusivity (MD), axial diffusivity (AD) and radial diffusivity (RD). MD is related to AD and RD through the following formula:

$$MD=\frac{1}{3}AD+\frac{2}{3}RD$$

We then applied tract-based spatial statistics (TBSS)^20^ and aligned all images to the FMRIN58_FA standard-space^58^ image. Subject FA-images were skeletonised using the IIT Human Brain Atlas (v.5.0)^59^ templates (IIT-FA-skeleton) and the IIT variation of the TBSS-script. A template rather than a study derived skeleton was chosen due to the concerns about pathological influences on the projection to the skeleton^19^. These concerns cannot entirely be eliminated but are limited to a subject-level rather than the group-level. For the non-FA parameters, we reused the non-linear registration from the FA-images to the skeletonised map with the IIT version of the TBSS script.

The lesion maps, the volume of lesions and the volume of white matter (WM) were obtained using the ico**brain** 3.1 software, previously known as MSmetrix^60^. The lesion maps and the diffusion tensor maps were transformed into the MNI space with the ANTS registration toolbox^61^ and using the 2 mm standard template available in FSL as reference^58^.

### Statistical analysis

In a first analysis, we looked at the differences between PwMS and HS by performing a nonparametric permutation, using the FSL randomise tool^62^ with 2000 permutations and the Threshold-Free Cluster Enhancement (TFCE) option, which generates results that are corrected for multiple comparisons across space. A threshold of 0.05 for statistical significance was used for the corrected p-value. This resulted in a binary 3D image indicating the spatial regions in which the tensor parameters are significantly different between PwMS and HS.

In a second analysis, we assessed possible associations between tensor parameters and neuropsychological tests and the association between tensor parameters and the lesion and WM volume. We used a general linear model: the neuropsychological test z-scores were used as weights in the design matrix of TBSS and similarly to the first analysis, we obtained the significant clusters with TFCE and used a threshold for the corrected p-values of 0.05, which we further lowered to 0.001 to account for the multiple comparisons (as each tensor parameter was compared to six parameters).

However, the cluster enhancement technique gains spatial specificity at the cost of losing all the information about the cluster strength. For this reason, we added a cluster analysis and used a voxel-wise analysis within the found clusters to gain information about the strength of these clusters. Associations between neuropsychological test z-scores and the values of the skeletonized tensor parameters were assessed through Pearson’s correlations in each voxel of the clusters.

As the last step of the second analysis, we used an atlas of white matter bundles, the IIT Human Brain Atlas (v.5.0)^59^, and retained the strongest correlation (highest r-value) with a p-value lower than 0.001 (to compensate for multiple comparisons) for each bundle in the atlas. The second analysis was carried out for HS and PwMS together, as we assumed that the correlation effect is independent of the presence of disease. Furthermore, by including both HS and PwMS, we can assess the correlation between DTI measures and cognition in a sample with a wide range of cognitive performances. The analysis was performed for only the PwMS as well but will only be included in the repository (https://neurovault.org/collections/LYEGOWBT/) and will not be discussed in this manuscript.

### Permission to reproduce material from other sources

All illustrations are derived from the data used in this paper and were created only for this manuscript with the FSLeyes toolbox or in MATLAB (2021). No material was copied from another source.

## Data Availability

Public sharing of the data used in this manuscript is not possible due to privacy and ethical restrictions. The data that support the findings of this study are available on request to the corresponding author.
